# Performance Assessment of the Universal Vital Assessment Score vs Other Illness Severity Scores for Predicting Risk of In-Hospital Death Among Adult Febrile Inpatients in Northern Tanzania, 2016-2019

**DOI:** 10.1001/jamanetworkopen.2021.36398

**Published:** 2021-12-16

**Authors:** John P. Bonnewell, Matthew P. Rubach, Deng B. Madut, Manuela Carugati, Michael J. Maze, Kajiru G. Kilonzo, Furaha Lyamuya, Annette Marandu, Nathaniel H. Kalengo, Bingileki F. Lwezaula, Blandina T. Mmbaga, Venance P. Maro, John A. Crump

**Affiliations:** 1 Department of Pathology, Duke University Medical Center, Durham, North Carolina; 2 Division of Infectious Diseases and International Health, Department of Medicine, Duke University Medical Center, Durham, North Carolina; 3 Duke Global Health Institute, Duke University, Durham, North Carolina; 4 Kilimanjaro Christian Medical Centre, Moshi, Tanzania; 5 Programme in Emerging Infectious Diseases, Duke–National University of Singapore Medical School, Singapore; 6 Department of Medicine, University of Otago, Dunedin, New Zealand; 7 Centre for International Health, University of Otago, Dunedin, New Zealand; 8 Kilimanjaro Christian Medical University College, Tumaini University, Moshi, Tanzania; 9 Mawenzi Regional Referral Hospital, Moshi, Tanzania; 10 Kilimanjaro Clinical Research Institute, Moshi, Tanzania

## Abstract

**Importance:**

Severity scores are used to improve triage of hospitalized patients in high-income settings, but the scores may not translate well to low- and middle-income settings such as sub-Saharan Africa.

**Objective:**

To assess the performance of the Universal Vital Assessment (UVA) score, derived in 2017, compared with other illness severity scores for predicting in-hospital mortality among adults with febrile illness in northern Tanzania.

**Design, Setting, and Participants:**

This prognostic study used clinical data collected for the duration of hospitalization among patients with febrile illness admitted to Kilimanjaro Christian Medical Centre or Mawenzi Regional Referral Hospital in Moshi, Tanzania, from September 2016 through May 2019. All adult and pediatric patients with a history of fever within 72 hours or a tympanic temperature of 38.0 °C or higher at screening were eligible for enrollment. Of 3761 eligible participants, 1132 (30.1%) were enrolled in the parent study; of those, 597 adults 18 years or older were included in this analysis. Data were analyzed from December 2019 to September 2021.

**Exposures:**

Modified Early Warning Score (MEWS), National Early Warning Score (NEWS), quick Sequential Organ Failure Assessment (qSOFA), Systemic Inflammatory Response Syndrome (SIRS) assessment, and UVA.

**Main Outcomes and Measures:**

The main outcome was in-hospital mortality during the same hospitalization as the participant’s enrollment. Crude risk ratios and 95% CIs for in-hospital death were calculated using log-binomial risk regression for proposed score cutoffs for each of the illness severity scores. The area under the receiver operating characteristic curve (AUROC) for estimating the risk of in-hospital death was calculated for each score.

**Results:**

Among 597 participants, the median age was 43 years (IQR, 31-56 years); 300 participants (50.3%) were female, 198 (33.2%) were HIV-infected, and in-hospital death occurred in 55 (9.2%). By higher risk score strata for each score, compared with lower risk strata, risk ratios for in-hospital death were 3.7 (95% CI, 2.2-6.2) for a MEWS of 5 or higher; 2.7 (95% CI, 0.9-7.8) for a NEWS of 5 or 6; 9.6 (95% CI, 4.2-22.2) for a NEWS of 7 or higher; 4.8 (95% CI, 1.2-20.2) for a qSOFA score of 1; 15.4 (95% CI, 3.8-63.1) for a qSOFA score of 2 or higher; 2.5 (95% CI, 1.2-5.2) for a SIRS score of 2 or higher; 9.1 (95% CI, 2.7-30.3) for a UVA score of 2 to 4; and 30.6 (95% CI, 9.6-97.8) for a UVA score of 5 or higher. The AUROCs, using all ordinal values, were 0.85 (95% CI, 0.80-0.90) for the UVA score, 0.81 (95% CI, 0.75-0.87) for the NEWS, 0.75 (95% CI, 0.69-0.82) for the MEWS, 0.73 (95% CI, 0.67-0.79) for the qSOFA score, and 0.63 (95% CI, 0.56-0.71) for the SIRS score. The AUROC for the UVA score was significantly greater than that for all other scores (*P* < .05 for all comparisons) except for NEWS (*P* = .08).

**Conclusions and Relevance:**

This prognostic study found that the NEWS and the UVA score performed favorably compared with other illness severity scores in predicting in-hospital mortality among a hospitalized cohort of adults with febrile illness in northern Tanzania. Given its reliance on readily available clinical data, the UVA score may have utility in the triage and prognostication of patients admitted to the hospital with febrile illness in low- to middle-income settings such as sub-Saharan Africa.

## Key Points

**Question:**

How does the Universal Vital Assessment (UVA) score perform compared with other common illness severity scores in predicting in-hospital death for febrile hospitalized patients in a lower-income setting?

**Findings:**

In this prognostic study of 597 adults hospitalized with febrile illness in northern Tanzania, the UVA score and National Early Warning Score outperformed other illness severity scores at predicting risk of in-hospital death. Scores of 2 or higher on the UVA were associated with significant increases in risk of death.

**Meaning:**

The findings of this study indicate that, as a clinical illness severity measure using readily available clinical data appropriate to resource-limited settings, the UVA shows promise for predicting risk of in-hospital mortality among febrile inpatients in lower-income settings.

## Teaser

This prognostic study compares the performance of the Universal Vital Assessment with that of other common illness severity tests for predicting the risk of in-hospital death among febrile hospitalized patients in northern Tanzania.

## Introduction

Infectious diseases are a leading cause of acute illness, disability, and death worldwide.^1,2^ Despite efforts to improve scientific knowledge of febrile illness in low- and middle-income countries (LMICs),^3,4,5,6,7^ the tools available to improve management of such acute illness remain inadequate.

A major component of research on the management of acute febrile illness focuses on the appropriate identification and triage of severely ill patients once they reach the health care system. In high-income countries, several illness severity scores have been developed for patients with severe illness, including patients with febrile illness.^8,9,10^ However, most such models were developed entirely within high-income settings. In sub-Saharan Africa and other LMIC settings, the etiology of febrile illness differs from that in temperate high-income countries.^3,5,6^ Furthermore, severe febrile illness outcomes likely differ owing to patterns of comorbidities and the availability of intensive care.^11^ It is unsurprising that studies of existing illness severity scores derived in high-income countries have shown mixed results for predictive performance for adverse outcomes in LMICs.^12,13,14,15,16^

In response to the limitations of existing predictive outcome models to identify hospital-admitted patients at high risk of death in resource-limited settings, the Universal Vital Assessment (UVA) score was derived and initially proposed in 2017 to predict inpatient mortality in sub-Saharan Africa.^17^ Derived from pooled data from studies of hospitalized patients in sub-Saharan Africa, the score uses easily measured clinical data, including temperature, heart and respiratory rates, systolic blood pressure, oxygen saturation, Glasgow Coma Scale scores, and HIV infection status.^17^ Initial internal validation showed a greater area under the receiver operating characteristic curve (AUROC) than that for the Modified Early Warning Score (MEWS)^18^ and the quick Sequential Organ Failure Assessment (qSOFA) score^8^ in the pooled cohort. To our knowledge, to date, 3 prospective external assessments have compared UVA scores with other predictive scores in sub-Saharan Africa: 1 showed only moderate performance across scores for predicting mortality at a single center in Rwanda^19^; another showed poor performance in a single-center, primarily surgical intensive care cohort in Malawi^20^; and a study in Gabon showed favorable performance for the UVA but was markedly limited by sample size and statistical power.^21^

We assessed the predictive performance of the UVA score for in-hospital mortality within a prospective cohort of patients admitted with febrile illness to 1 of 2 hospitals in northern Tanzania. We also compared the performance of the UVA with that of other common predictive tools—the National Early Warning Score (NEWS),^22^ the MEWS, the qSOFA score, and the Systemic Inflammatory Response Syndrome (SIRS) score.^9^

## Methods

### Ethics Statement

This prognostic study was approved by the Kilimanjaro Christian Medical University College’s Research Ethical Review Committee, the United Republic of Tanzania’s National Institute for Medical Research National Health Research Ethics Committee, and the Duke University Health System Institutional Review Board. Adults 18 years or older provided their own written consent when feasible, and those with impaired consciousness were enrolled with written consent from next of kin. This study was conducted in accordance with the Standards for Reporting of Diagnostic Accuracy (STARD) 2015 reporting guideline (eTable 2 in the Supplement).^23^

### Study Setting

Moshi is a municipality (population approximately 200 000) and the administrative center of the Kilimanjaro Region (population >1.6 million) in northern Tanzania. In 2016-2017, the estimated prevalence of HIV among adults 15 to 64 years in the Kilimanjaro Region was 2.6%.^24^ Viral suppression of HIV in the Kilimanjaro Region was estimated at 67% in 2017 among individuals who access health care resources.^24^ The transmission intensity of malaria is low in the Kilimanjaro Region,^25^ with an estimated child community malaria parasite prevalence of less than 1% in 2017.^26^ Kilimanjaro Christian Medical Centre is the 630-bed referral hospital for northern Tanzania and the site of a university medical college. Mawenzi Regional Referral Hospital is a 300-bed regional hospital that serves the Kilimanjaro Region.

### Study Design and Procedures

We conducted a prospective cohort study of patients with febrile illness admitted to Kilimanjaro Christian Medical Centre or Mawenzi Regional Referral Hospital in Moshi, Tanzania, from September 2016 to May 2019. Both adult and pediatric patients were included in the study. For the data analysis of the present prognostic study, participants were included if 18 years or older, consistent with the initial derivation and validation of the UVA score.^17^ Within 24 hours of admission, all adult and pediatric patients admitted to the medical ward were screened for those with a history of fever within 72 hours or a tympanic temperature of 38.0 °C or higher. Enrolled participants had a standardized clinical history and physical examination performed by trained study staff that included measurement of vital signs, mid–upper arm circumference,^27^ percutaneous oximetry, and assessment of mental status by both the Glasgow Coma Scale and alert-voice-pain-unconscious scores. Blood testing was performed, including HIV antibody testing, blood cultures, complete blood cell counts, blood smear for parasites, and lactate testing. Testing for HIV antibodies was performed using SD BIOLINE HIV 1/2 3.0 (Abbott Laboratories), and positive tests were confirmed by Uni-Gold HIV (Trinity Biotech). Blood culture bottles were BACT/ALERT FA PLUS bottles loaded into the BACT/ALERT 3D microbial detection system (bioMérieux), and 2 sets of culture bottles were collected when feasible. Testing for CD4^+^ T lymphocytes was performed for HIV-infected participants using the FACSCalibur platform (BD Biosciences). Lactate testing was performed using the StatStrip Lactate System (Nova Biomedical). Participants were followed up during their hospitalization for ascertainment of in-hospital outcome.

### Definitions

The primary outcome for this study was in-hospital death, defined as participant death at Kilimanjaro Christian Medical Centre or Mawenzi Regional Referral Hospital during the same hospitalization as their enrollment. Blood cultures were considered positive if there was growth from either set of cultures collected. Single cultures positive for normal skin flora, including *Micrococcus* species, coagulase-negative *Staphylococcus* species, and *Streptococcus* species other than *S pneumoniae*, were not considered to be potential pathogens. Hypoxemia was defined as peripheral oxygen saturation of less than 92% on room air or the use of supplemental oxygen. A lactate level cutoff of 36 mg/dL or more (to convert to millimoles per liter, multiply by 0.111) was chosen in the analysis given its greater likelihood of representing participants with shock than a cutoff of 18 mg/dL or more; both thresholds were assessed in the Third International Consensus Definitions for Sepsis and Septic Shock definition.^8^

Scores for predictive models were determined based on the original definitions proposed for each model, using clinical data collected by the study team at enrollment.^8,10,18,22^ The full calculation scheme for each severity score is depicted in eTable 1 in the Supplement. All scores were analyzed by standard proposed cutoffs and in their entirety, including all ordinal values, with respect to performance characteristics in the study cohort. Although all scores included in this study have been assessed for performance in predicting inpatient mortality,^8,16,17,18,22^ the MEWS and NEWS are primarily used to assess the risk of inpatient clinical deterioration,^18,22^ and the qSOFA and SIRS scores have been used to assist in both classifying and triaging patients with sepsis.^8,9^

Participants were also classified based on the World Health Organization’s Integrated Management of Adolescent and Adult Illness guidelines^28^ as having septic shock or severe respiratory distress without shock. Septic shock was defined by the Integrated Management of Adolescent and Adult Illness guidelines as systolic blood pressure less than 90 mm Hg and either a heart rate greater than 100/min, a respiratory rate greater than 24/min, or a temperature greater than 38 °C or less than 36 °C. The Integrated Management of Adolescent and Adult Illness guidelines defined severe respiratory distress without shock as a respiratory rate greater than 30/min or peripheral oxygen saturation less than 90% and a systolic blood pressure of 90 mm Hg or greater. Presence of radiographic pneumonia or clinical heart failure were excluded from the latter definition because these data were not available.

### Power Calculation

We performed an assessment of statistical power for this study based on our ability to find a difference between the UVA and qSOFA scores as the primary comparators of interest. Based on published literature, we expected AUROC values for predicting in-hospital mortality of 0.85 for the UVA and 0.75 for the qSOFA.^17,21,29^ With an expected mortality of 10% based on a prior febrile cohort at our 2 study hospitals^3^ and assuming at least moderate correlation between UVA and qSOFA scores (*r* ≥ 0.4) for the 597 participants included in the study, we estimated that our analysis had a minimum of 80% power to detect a difference of 0.1 between AUROC values for UVA and qSOFA scores.

### Statistical Analysis

Descriptive statistics were used, including medians and IQRs for nonnormally distributed variables, as assessed by the Shapiro-Wilk test. No normally distributed continuous variables were included. Categorical variables were presented as frequencies. To assess for differences between those who died in the hospital and those who survived, the rank sum test was used for nonnormally distributed continuous variables. The χ^2^ test of independence was used to compare proportions for binomial variables where all observed counts of cells in a 2 × 2 contingency table were 5 or greater; the Fisher exact test was used for any with fewer than 5. Crude risk ratios and 95% CIs for in-hospital death were calculated using log-binomial risk regression for each proposed score cutoff. Performance characteristics for predicting in-hospital death were calculated for each score, which included sensitivity, specificity, positive and negative predictive values, positive and negative likelihood ratios, and the AUROC. The AUROCs were compared using a method suggested by DeLong et al^30^ as implemented in Stata, version 16 (StataCorp LLC). Statistical significance was defined as 2-sided *P* < .05 for each of these comparisons. All statistical analyses were performed using Stata, version 16. Data were analyzed from December 2019 to September 2021.

## Results

### Baseline Clinical Characteristics

**Figure 1.**
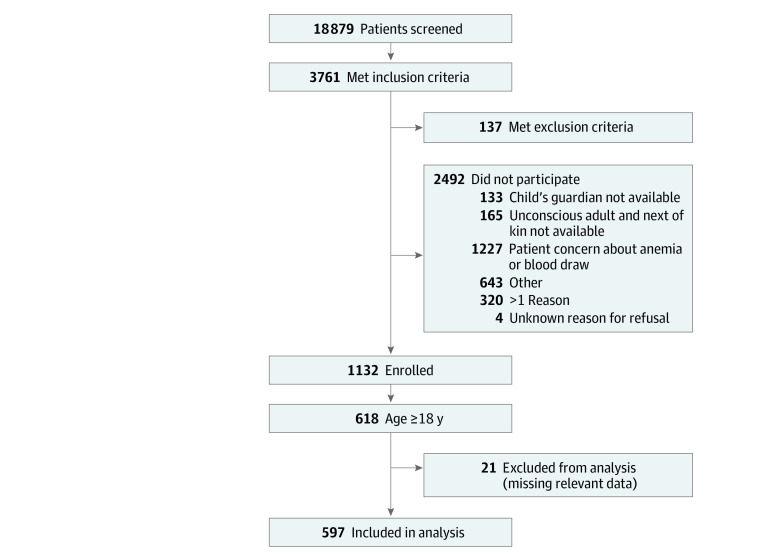
Enrollment Flowchart for Adults Seen in the Hospital for Febrile Illness, Northern Tanzania, 2016-2019

Of 3761 eligible participants, 1132 (30.1%) were enrolled in the parent study, 618 of whom (54.6%) were adults 18 years or older and eligible for the present analysis (Figure 1). Of the 618 eligible participants, 21 (3.4%) were excluded owing to missing variables relevant to score calculation; thus, 597 adults were included in the final analysis. The median age of participants was 43 years (IQR, 31-56 years) (*P* = .08), and 300 participants (50.3%) were female (*P* = .13).

Baseline characteristics from the full cohort and the HIV-infected subgroup by outcome are compared in Table 1. Of the 597 participants, 55 (9.2%) died in the hospital. There were 198 HIV-infected participants (33.2% of the full cohort), of whom 35 (17.7%) died in the hospital. Thirty-seven HIV-infected patients (18.7%) reported no HIV infection at the time of admission and tested positive for HIV during their hospitalization. The median CD4^+^ T lymphocyte count among all participants with an HIV infection was 121 cells/mm^3^ (IQR, 24-288 cells/mm^3^). Among participants who reported no HIV infection but tested positive for HIV, the median CD4^+^ T lymphocyte count was 138 cells/mm^3^ (IQR, 46-230 cells/mm^3^). Of the 161 participants who reported HIV infection at the time of enrollment, 120 (74.5%) reported taking any antiretroviral regimen and 95 (59.0%) reported taking trimethoprim-sulfamethoxazole.

**Table 1.** Baseline Characteristics of Participants Admitted to the Hospital With Febrile Illness, Northern Tanzania, 2016-2019

Variable	Full cohort				HIV-infected cohort			
	Patients, No. (%)^a^			*P* value	Patients, No. (%)^a^			*P* value
	Total (N = 597)	Survivors (n = 542)	In-hospital deaths (n = 55)		Total (N = 198)	Survivors (n = 163)	In-hospital deaths (n = 35)	
General demographic characteristics^b^								
Age, median (IQR), y	43 (31-56)	43 (30-56)	44 (36-59)	.08	43 (34-48)	42 (34-48)	43 (34-49)	.77
Female	300 (50.3)	267 (49.3)	33 (60.0)	.13	121 (61.1)	98 (60.1)	23 (65.7)	.54
Male	297 (49.7)	275 (50.7)	22 (40.0)	.13	77 (38.9)	65 (39.9)	12 (34.3)	.54
Duration of illness, median (IQR), d^c^	6 (3-14)	5 (3-14)	12 (4-21)	.58	7 (4-21)	7 (4-21)	14 (4-21)	.88
Clinical characteristics								
MUAC, median (IQR), cm^d^	26.6 (24.0-30.0)	27.0 (24.0-30.0)	25.0 (22.0-27.8)	<.001	25.0 (23.0-27.5)	25.3 (23.0-28.0)	24.5 (20.5-26.0)	.01
Temperature >38 °C	267 (44.7)	235 (43.4)	32 (58.1)	.04	100 (50.5)	80 (49.1)	20 (57.1)	.39
Temperature <36 °C	8 (1.3)	6 (1.1)	2 (3.6)	.16	5 (2.5)	3 (1.8)	2 (5.7)	.21
Heart rate >90/min	359 (60.1)	314 (57.9)	45 (81.8)	.01	145 (73.2)	115 (70.6)	30 (85.7)	.07
Respiratory rate >20/min	398 (66.7)	356 (65.7)	42 (76.4)	.11	135 (68.2)	110 (67.5)	25 (71.4)	.65
SBP <90 mm Hg	32 (5.4)	27 (5.0)	5 (9.1)	.20	14 (7.1)	11 (6.8)	3 (8.6)	.72
GCS score <15	110 (18.4)	77 (14.2)	33 (60.0)	<.001	45 (22.7)	25 (15.3)	20 (57.1)	<.001
Hypoxemia	110 (18.4)	84 (15.5)	26 (47.3)	<.001	43 (21.7)	31 (19.0)	12 (34.3)	.05
IMAI septic shock	17 (2.9)	13 (2.4)	4 (7.3)	.06	9 (4.6)	7 (4.3)	2 (5.7)	.66
IMAI severe respiratory distress without shock	99 (16.6)	69 (12.7)	30 (54.6)	<.001	37 (18.7)	21 (12.9)	16 (45.7)	<.001
Laboratory results								
WBC count >12 000/μL	112 (18.8)	99 (18.3)	13 (23.6)	.33	28 (14.1)	23 (14.1)	5 (14.3)	.98
WBC count <4000/μL × 10^9^/L	111 (18.6)	103 (19.0)	8 (14.6)	.42	55 (27.8)	49 (30.1)	6 (17.1)	.12
Lactate level ≥36 mg/dL	22 (4.6)	17 (3.9)	5 (12.8)	.01	12 (7.7)	8 (6.2)	4 (16.0)	.11
Pathogen detected by blood culture	45 (7.5)	33 (6.1)	12 (21.8)	<.001	18 (9.1)	10 (6.1)	8 (22.9)	.002
Positive malaria smear	34 (5.7)	34 (6.3)	0	NA	3 (1.5)	3 (1.8)	0	NA
CD4^+^ T-cell count, median (IQR), cells/mm^3^	NA	NA	NA	NA	121 (24-288)	149 (32-355)	29.5 (12.5-124)	.001

Abbreviations: GCS, Glasgow Coma Scale; IMAI, Integrated Management of Adolescent and Adult Illness; MUAC, mid–upper arm circumference; NA, not applicable; SBP, systolic blood pressure; WBC, white blood cell.

SI conversion factors: To convert the WBC count to cells ×10^9^/L, multiply by 0.001; lactate level to millimoles per liter, multiply by 0.111.

^a^ Data are presented as the number (percentage) of patients unless otherwise indicated.

^b^ Values may not sum to the total number of patients for some variables owing to missing data.

^c^ Duration of illness was the duration, in days, of symptoms before enrollment as reported by the participants or their representatives.

^d^ MUAC has been used as an indicator of low body mass index and malnutrition, particularly in children; however, no universal cutoff exists for adults. A cutoff of 24.0 cm or less for adults has been proposed by the US Agency for International Development’s Food and Nutrition Technical Assistance III Project.^27^

### Baseline Prognostic Scores for Participants

The median scores for each prognostic test and the risk of death based on proposed cutoff scores by prognostic test for the total cohort and the HIV-infected cohort are summarized in Table 2. For the total cohort by UVA score, compared with participants with scores less than 1, the RRs for in-hospital death were 9.1 (95 CI, 2.7-30.3) for those with scores from 2 to 4 and 30.6 (95% CI, 9.6-97.8) for those with scores of 5 or higher. For the NEWS, compared with the low-risk group (scores ≤4), the RRs were 2.7 (95% CI, 0.9-7.8) for those with scores of 5 or 6 and 9.6 (95% CI, 4.2-22.2) for those with scores of 7 or higher. For the qSOFA, compared with participants with scores of 0, those with scores of 1 had an RR of 4.8 (95% CI, 1.2-20.2) compared with 15.4 (95% CI, 3.8-63.1) for those with scores of 2 or higher. For the dichotomous risk strata of the 2 other tests, compared with the lower-risk groups, the RRs for death were 2.5 (95% CI, 1.2-5.2) for those with a SIRS score of 2 or higher and 3.7 (95% CI, 2.2-6.2) for those with a MEWS of 5 or higher.

**Table 2.** Baseline Risk Scores and Risk of Death by Proposed Risk Score Cutoffs for Participants With Febrile Illness, Northern Tanzania, 2016-2019

Prognostic test score^a^	Full cohort				HIV-infected cohort			
	Patients, No. (%)^b^			Risk ratio (95% CI)	Patients, No. (%)^b^			Risk ratio (95% CI)
	Total (N = 597)	Survivors (n = 542)	In-hospital deaths (n = 55)		Total (N = 198)	Survivors (n = 163)	In-hospital deaths (n = 35)	
MEWS								
Median (IQR)	3 (2-5)	3 (2-5)	5 (4-7)	NA	4 (2-6)	4 (2-5)	6 (4-7)	NA
≤4	415 (69.5)	394 (72.7)	21 (38.2)	1 [Reference]	117 (59.1)	105 (64.4)	12 (34.3)	1 [Reference]
≥5	182 (30.5)	148 (27.3)	34 (61.8)	3.7 (2.2-6.2)	81 (40.9)	58 (35.6)	23 (65.7)	2.8 (1.5-5.2)
NEWS								
Median (IQR)	5 (3-7)	5 (3-7)	9 (7-13)	NA	6 (2-6)	4 (2-5)	6 (4-7)	NA
≤4	276 (46.2)	270 (49.8)	6 (10.9)	1 [Reference]	58 (29.3)	54 (33.1)	4 (11.4)	1 [Reference]
5-6	120 (20.1)	113 (20.9)	7 (12.7)	2.7 (0.9-7.8)	45 (22.7)	41 (25.2)	4 (11.4)	1.3 (0.3-4.9)
≥7	201 (33.7)	159 (29.3)	42 (76.4)	9.6 (4.2-22.2)	95 (48.0)	68 (41.7)	27 (77.1)	4.1 (1.5-11.2)
qSOFA								
Median (IQR)	1 (1-1)	1 (0-1)	2 (1-2)	NA	1 (1-2)	1 (1-2)	1 (1-2)	NA
0	139 (23.3)	137 (25.3)	2 (3.6)	1 [Reference]	35 (17.7)	33 (20.3)	2 (5.7)	1 [Reference]
1	318 (53.3)	296 (54.6)	22 (40.0)	4.8 (1.2-20.2)	102 (51.5)	88 (54.0)	14 (40.0)	2.4 (0.6-10.0)
≥2	140 (23.5)	109 (20.1)	31 (56.4)	15.4 (3.8-63.1)	61 (30.8)	42 (25.8)	19 (54.3)	5.5 (1.3-22.0)
SIRS								
Median (IQR)	2 (1-3)	2 (1-3)	3 (2-3)	NA	2 (2-3)	2 (2-3)	2 (2-3)	NA
≤1	178 (29.8)	170 (31.4)	8 (14.6)	1 [Reference]	35 (17.7)	31 (19.0)	4 (11.4)	1 [Reference]
≥2	419 (70.2)	372 (68.6)	47 (85.5)	2.5 (1.2-5.2)	163 (82.3)	132 (81.0)	31 (88.6)	1.7 (0.6-4.4)
UVA								
Median (IQR)	2 (0-4)	1 (0-3)	6 (3-7)	NA	3 (2-6)	2 (2-4)	6 (3-8)	NA
≤1	287 (48.1)	284 (52.4)	3 (5.5)	1 [Reference]	0	0	0	NA
2-4	210 (35.2)	190 (35.1)	20 (36.4)	9.1 (2.7-30.3)	137 (69.2)	124 (76.1)	13 (37.1)	1 [Reference]
≥5	100 (16.8)	68 (12.6)	32 (58.2)	30.6 (9.6-97.8)	61 (30.8)	39 (23.9)	22 (62.9)	3.8 (2.1-7.0)

Abbreviations: MEWS, Modified Early Warning Score; NA, not applicable; NEWS, National Early Warning Score; qSOFA, quick Sequential Organ Failure Assessment; SIRS, Systemic Inflammatory Response Syndrome; UVA, Universal Vital Assessment.

^a^ Percentages in the columns of total patients are the proportions of the total number of patients within each score range for each prognostic score. Percentages of survivors and in-hospital deaths are the percentages of patients who survived or died within that specific score range rather than of the total number of patients for each column.

^b^ Data are presented as the number (percentage) of patients unless otherwise indicated.

### Performance of Predictive Scores

The sensitivity of the respective scores and their cutoffs for predicting death ranged from 56.4% to 96.4%, whereas specificity had a range of 25.3% to 87.5% (Table 3). Positive predictive values ranged from 11.2% to 32.0%, and negative predictive values ranged from 94.7% to 99.0%. Moving the UVA cutoff from a score of 2 or higher to a score of 5 or higher was associated with a decrease in sensitivity from 94.5% (95% CI, 84.0%-98.9%) to 58.2% (95% CI, 44.1%-71.3%) and an increase in specificity from 52.4% (95% CI, 48.1%-56.7%) to 87.5% (95% CI, 84.4%-90.1%).

**Table 3.** Risk Score Prognostic Performance for In-Hospital Death by Proposed Score Cutoffs in Participants With Febrile Illness, Northern Tanzania, 2016-2019

Test and score cutoff	Sensitivity, % (95% CI)	Specificity, % (95% CI)	Positive predictive value^a^	Negative predictive value^a^	Positive likelihood ratio (95% CI)	Negative likelihood ratio (95% CI)	AUROC (95% CI)
MEWS ≥5	61.8 (47.7-74.6)	72.7 (68.7-76.4)	18.7 (13.3-25.1)	94.9 (92.4-96.8)	2.26 (1.76-2.90)	0.53 (0.37-0.74)	0.67 (0.61-0.74)
NEWS ≥5	89.1 (77.8-95.9)	49.8 (45.5-54.1)	15.3 (11.5-19.7)	97.8 (95.3-99.2)	1.78 (1.57-2.01)	0.22 (0.10-0.47)	0.70 (0.65-0.74)
NEWS ≥7	76.4 (63.0-86.8)	70.7 (66.6-74.5)	20.9 (15.5-27.2)	96.7 (94.5-98.2)	2.60 (2.14-3.17)	0.33 (0.21-0.54)	0.74 (0.68-0.80)
qSOFA ≥1	96.4 (87.5-99.6)	25.3 (21.7-29.2)	11.6 (8.8-14.9)	98.6 (94.9-99.8)	1.29 (1.20-1.38)	0.14 (0.04-0.57)	0.61 (0.58-0.64)
qSOFA ≥2	56.4 (42.3-69.7)	79.9 (76.3-83.2)	22.1 (15.6-29.9)	94.7 (92.3-96.6)	2.80 (2.10-3.73)	0.55 (0.40-0.74)	0.68 (0.40-0.74)
SIRS ≥2	85.5 (73.3-93.5)	31.4 (27.5-35.5)	11.2 (8.4-14.6)	95.5 (91.3-98.0)	1.25 (1.10-1.41)	0.46 (0.24-0.89)	0.58 (0.52-0.64)
UVA ≥2	94.5 (84.0-98.9)	52.4 (48.1-56.7)	16.8 (12.8-21.4)	99.0 (97.0-99.8)	1.99 (1.78-2.21)	0.10 (0.03-0.31)	0.74 (0.70-0.77)
UVA ≥5	58.2 (44.1-71.3)	87.5 (84.4-90.1)	32.0 (23.0-42.1)	95.4 (93.1-97.0)	4.64 (3.38-6.36)	0.48 (0.35-0.65)	0.73 (0.66-0.80)

Abbreviations: AUROC, area under the receiver operating characteristic curve; MEWS, Modified Early Warning Score; NEWS, National Early Warning Score; qSOFA, quick Sequential Organ Failure Assessment; SIRS, Systemic Inflammatory Response Syndrome; UVA, Universal Vital Assessment.

^a^ Calculated using an in-hospital death outcome prevalence of 9.2% (95% CI, 7.0%-11.8%) as noted in the overall cohort in this study.

**Figure 2.**
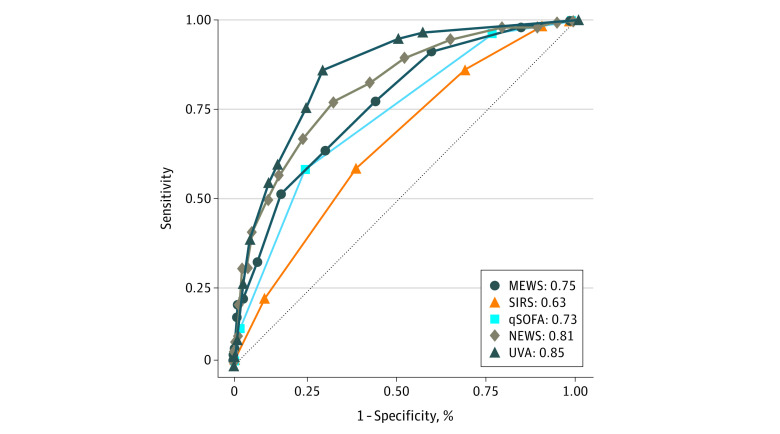
Area Under the Receiver Operating Characteristic Curve (AUROC) Values for Clinical Prognostic Scores Using All Ordinal Values for In-Hospital Death in Participants With Febrile Illness, Northern Tanzania, 2016-2019 The dashed diagonal line is a reference line representative of a test with no discriminatory ability (AUROC = 0.5). MEWS indicates Modified Early Warning Score; NEWS, National Early Warning Score; qSOFA, quick Sequential Organ Failure Assessment; SIRS, systemic inflammatory response syndrome; and UVA, Universal Vital Assessment.

The AUROC values by severity score cutoff are also shown in Table 3. The AUROC values ranged from 0.58 for a SIRS score of 2 or higher to 0.74 for both a UVA score of 2 or higher and a NEWS of 7 or higher. Considering performance of the scores by all included ordinal values, the AUROC values were as follows (Figure 2): UVA, 0.85 (95% CI, 0.80-0.90); NEWS, 0.81 (95% CI, 0.75-0.87); MEWS, 0.75 (95% CI, 0.69-0.82); qSOFA, 0.73 (95% CI, 0.67-0.79); and SIRS, 0.63 (95% CI, 0.56-0.71). When these AUROC values were compared with one another, UVA scores had statistically higher values than MEWS, qSOFA, and SIRS scores (*P* < .001 for each) but not NEWS (*P* = .08). Scores for the participants with an HIV infection are presented in eTables 3 and 4 in the Supplement.

## Discussion

In this prognostic study of illness severity scores among hospitalized adult patients with febrile illness, the UVA score and NEWS showed the best performance for estimating the risk of in-hospital death among the study cohort, with AUROC values of 0.85 and 0.81, respectively. The AUROC values decreased for the MEWS, qSOFA score, and SIRS score, respectively. Most of the cutoffs for each prognostic score were statistically associated with an increased risk of in-hospital death with increasing score. However, the UVA score showed the greatest discriminatory power, with statistically significant and meaningful increases in estimated risk of death for scores of 2 to 4 and 5 or higher; NEWS values of 5 to 6 compared with those of 4 or lower were not associated with a significant increase in mortality.

These data are consistent with the findings of derivation and validation studies of the UVA score’s predictive performance compared with that of other illness severity scores in LMICs.^17,21^ In the UVA derivation study, among participants with suspected infection, the AUROC for the UVA was the greatest (0.75; 95% CI, 0.72-0.77) compared with that of the MEWS (0.65; 95% CI, 0.63-0.68) and the qSOFA (0.68; 95% CI, 0.65-0.71).^17^ The prognostic score performance data also are consistent with the findings from a prospective validation study from Gabon, in which the UVA score had an AUROC of 0.90 (95% CI, 0.78-1.0) compared with that of the qSOFA score (0.77; 95% CI, 0.63-0.91) and the MEWS (0.72; 95% CI, 0.58-0.87) among 187 patients with suspected infection,^21^ although that study had limited statistical power to detect differences between the scores. The results of the current study differ from the findings of a study of 647 patients with suspected infection in Rwanda, in which the AUROC of the UVA score (0.71; 95% CI, 0.66-0.76) did not significantly differ from that of an adapted MEWS (0.69; 95% CI, 0.64-0.74) or the qSOFA score (0.65; 95% CI, 0.60-0.70).^19^ However, the Rwandan study and this study differed substantially in terms of inclusion criteria, enrollment (which in the Rwandan study was conducted throughout the course of hospitalization), and time points for data collection. In contrast, because we enrolled participants within 24 hours of hospital admission, our analysis assessed the performance of risk scores at a similar and important time period for febrile inpatients. This study’s data also differ from those of a Malawi study,^20^ wherein the AUROC for the UVA score was 0.54 (95% CI, 0.48-0.60). However, the Malawi study was notably different than the current study in that the cohort was smaller and primarily included participants requiring surgical intensive care, with all severity scores performing poorly in predicting outcomes.^20^ Overall, the current analysis provides additional support for use of the UVA score in patients in sub-Saharan Africa who are seen in the hospital with febrile illness, a common cause of hospital admission in LMIC settings.^1,31^

Other notable findings in this study include the limited performance of SIRS and qSOFA scores for estimating mortality risk in the study’s cohort of persons with fever. Both the SIRS criteria and the qSOFA were intended for the diagnosis and triage of patients with sepsis rather than prediction of in-hospital death. However, it is notable that SIRS criteria have since been abandoned in the most recent sepsis definition, in part owing to concerns about its poor specificity.^8^ The shortcomings of SIRS are reflected in this study’s findings of comparatively high sensitivity but poor specificity and poor positive predictive value for in-hospital death. For the qSOFA, despite this study’s finding of meaningful increases in risk of death with scores higher than 0, the limited performance based on the AUROC is consistent with the findings of other studies in LMICs.^29,32^

The NEWS and MEWS had moderate predictive performance in this study’s cohort. The MEWS has shown limited performance in prior studies in LMICs to date,^17,21^ but the NEWS has performed better in LMICs despite fewer publications assessing its potential utility in such settings.^33,34^ The performance of these tests is notable in that both scores were originally derived to predict inpatient clinical deterioration as opposed to predicting outcomes. However, both scores rely on frequent vital sign monitoring and are better suited to institutions with electronic health records that can provide real-time feedback on decompensation risk to prompt provider interventions, neither of which are available in many LMIC settings.^34^

### Strengths and Limitations

This study has several strengths. To our knowledge, it provides the first prospective external validation of the UVA score in a cohort of this size in which variables were assessed at a single time point early within a participant’s hospitalization.^1^ The use of a unified time point for clinical variables in this study’s cohort allowed for an assessment of the utility of the scores for early triage and prognostication on hospitalization. Furthermore, very few clinical data points were missing for score calculation. This not only allowed for improved assessment of the clinical scores but also allowed for a strong prospective validation of score performance for the large subset of participants with HIV infection (eTables 3 and 4 in the Supplement).

This study also has limitations. First is the limited generalizability of the data. The study was conducted at only 2 medical centers in northern Tanzania. Thus, demographic and epidemiological differences would likely be associated with different score performance elsewhere. Second, the strength of UVA and other scores is contingent on how they are used by clinicians, including whether a single cutoff score or the entire scoring system is used for risk stratification. For that reason, we presented data on each score for both proposed cutoffs and by all ordinal values. Further exploration of appropriate risk strata for each score is needed. Another limitation of the study is that relatively few eligible patients chose to participate in the study, which inherently risks introducing bias into this analysis. In addition, this study was conducted only with participants with febrile illness as opposed to a general population admitted to a medical ward. Further prospective validation of UVA scores is needed in populations with other common noninfectious conditions in LMICs.

## Conclusions

In this prognostic study of the performance of the UVA and other illness severity scores in a cohort of hospitalized patients with febrile illness in northern Tanzania, the UVA score showed favorable performance characteristics compared with other illness severity scores. Given the simplicity of calculation of the UVA score based on its reliance on readily available clinical parameters at a single time point, the UVA may be appropriate for triage and prognostication for hospitalized patients with febrile illness in LMIC settings such as sub-Saharan Africa.

## Notes

Supplement. **eTable 1.** Calculation Scheme for Risk Scores for Participants Presenting With Febrile Illness, Northern Tanzania, 2016-2019 **eTable 2.** Standards for Reporting Diagnostic Accuracy Studies (STARD) 2015 Reporting Guidelines **eTable 3.** Risk Score Prognostic Performance for In-Hospital Death by Proposed Cutoffs in HIV-Infected Participants With Febrile Illness, Northern Tanzania, 2016-2019 **eTable 4.** Risk Score Prognostic Performance by All Ordinal Values for In-Hospital Death in HIV-Infected Participants Admitted With Febrile Illness, Northern Tanzania, 2016-2019
