# Supplementary material for: Performance Assessment of the Universal Vital Assessment Score vs Other Illness Severity Scores for Predicting Risk of In-Hospital Death Among Adult Febrile Inpatients in Northern Tanzania, 2016-2019
Source: JAMA Netw Open. 2021 Dec 16;4(12):e2136398. doi: 10.1001/jamanetworkopen.2021.36398 (PMC8678687; doi:10.1001/jamanetworkopen.2021.36398)
Supplement: Supplement. — eTable 1. Calculation Scheme for Risk Scores for Participants Presenting With Febrile Illness, Northern Tanzania, 2016-2019 eTable 2. Standards for Reporting Diagnostic Accuracy Studies (STARD) 2015 Reporting Guidelines eTable 3. Risk Score Prognostic Performance for In-Hospital Death by Proposed Cutoffs in HIV-Infected Participants With Febrile Illness, Northern Tanzania, 2016-2019 eTable 4. Risk Score Prognostic Performance by All Ordinal Values for In-Hospital Death in HIV-Infected Participants Admitted With Febrile Illness, Northern Tanzania, 2016-2019 [file jamanetwopen-e2136398-s001.pdf]

Supplementary Online Content

Bonnewell JP, Rubach MP, Madut DB, et al. Performance assessment of the Universal Vital Assessment score vs other illness severity scores for predicting risk of in-hospital death among adult febrile inpatients in northern Tanzania, 2016-2019. *JAMA Netw Open*. 2021;4(12):e2136398. doi:10.1001/jamanetworkopen.2021.36398

eTable 1. Calculation Scheme for Risk Scores for Participants Presenting With Febrile Illness, Northern Tanzania, 2016-2019

eTable 2. Standards for Reporting Diagnostic Accuracy Studies (STARD) 2015 Reporting Guidelines

eTable 3. Risk Score Prognostic Performance for In-Hospital Death by Proposed Cutoffs in HIV-infected Participants With Febrile Illness, Northern Tanzania, 2016-2019

eTable 4. Risk Score Prognostic Performance by All Ordinal Values for In-Hospital Death in HIV-infected Participants Admitted With Febrile Illness, Northern Tanzania, 2016-2019

This supplementary material has been provided by the authors to give readers additional information about their work.

© 2021 Bonnewell JP et al. *JAMA Network Open*.

eTable 1. Calculation scheme for risk scores for participants presenting with febrile illness, northern Tanzania, 2016-2019.

MEWS[a] *(High risk: ≥5 points)*

Variable	3 points	2 points	1 point	0 points	1 point	2 points	3 points
RR (breaths/min)		≤8		9–14	15–20	21-29	>29
HR (beats/min)		≤40	41–50	51–100	101–110	111-129	>129
SBP (mmHg)	≤70	71–80	81–100	101–199		≥200	
Temperature (°C)		≤35.0	35.1–36.0	36.1–38.0	38.1–38.5	≥38.6	
AVPU scale				A	V	P	U

NEWS *(Medium risk: 5-6 points or any component variable of 3 points; High risk: ≥7 points)*

Variable	3 points	2 points	1 point	0 points	1 point	2 points	3 points
RR (breaths/min)	≤8		9-11	12-20		21-24	≥25
Oxygen saturation (%)	≤91	92-93	94-95	≥96			
Any supplemental oxygen		Yes		No			
Temperature (°C)	≤35.0		35.1-36.0	36.1-38.0	38.1-39.0	≥39.1	
SBP (mmHg)	≤90	91-100	101-110	111-219			≥220
HR (beats/min)	≤40		41-50	51-90	91-110	111-130	≥131
AVPU scale				A			V, P, or U

qSOFA score *(High risk: ≥2 points)*

Variable	0 points	1 point
GCS	15	<15
RR (breaths/min)	<22	≥22
SBP (mmHg)	>100	≤100

SIRS score *(High risk: ≥2 points)*

Variable	0 points	1 point
Temperature (°C)	36.0-38.0	>38.0 or <36.0
HR (beats/min)	≤90	>90
RR (breaths/min)	≤20	>20
WBC (cells/mm^3)	4,000-12,000	>12,000 or <4,000

UVA score *(Medium risk: 2-4 points; High risk: ≥5 points)*

Variable	0 points	1 point	2 points	3 points	4 points
Temperature (°C)	≥36.0		<36.0		
HR (beats/min)	<120	≥120			
RR (breaths/min)	<30	≥30			
SBP (mmHg)	≥90	<90			
Oxygen saturation (%)	≥92		<92		
GCS	15				<15
HIV infection	No/Unknown		Yes		

[a]Abbreviations: AVPU is Alert-Voice-Pain-Unresponsive; GCS is Glasgow Coma Scale; HR is heart rate; MEWS is Modified Early Warning System; qSOFA is quick Sequential Organ Failure Assessment; RR is respiratory rate; SBP is systolic blood pressure; SIRS is Systemic Inflammatory Response Syndrome; WBC is white blood cells.

© 2021 Bonnewell JP et al. *JAMA Network Open*.

eTable 2. Standards for reporting diagnostic accuracy studies (STARD) 2015 reporting guidelines.

Section and topic	No.	Item	Page no.
**TITLE OR ABSTRACT**	**1**	Identification as a study of diagnostic accuracy using at least one measure of accuracy (such as sensitivity, specificity, predictive values, or AUC)	1
**ABSTRACT**	**2**	Structured summary of study design, methods, results, and conclusions (for specific guidance, see STARD for Abstracts)	4
**INTRODUCTION**	**3**	Scientific and clinical background, including the intended use and clinical role of the index test	6-7
	**4**	Study objectives and hypotheses	7
**METHODS**			
Study design	**5**	Whether data collection was planned before the index test and reference standard were performed (prospective study) or after (retrospective study)	7-11
Participants	**6**	Eligibility criteria	8
	**7**	On what basis potentially eligible participants were identified (such as symptoms, results from previous tests, inclusion in registry)	8
	**8**	Where and when potentially eligible participants were identified (setting, location and dates)	8
	**9**	Whether participants formed a consecutive, random or convenience series	8
Test methods	**10a**	Index test, in sufficient detail to allow replication	eTable 1
	**10b**	Reference standard, in sufficient detail to allow replication	eTable 1
	**11**	Rationale for choosing the reference standard (if alternatives exist)	n/a
	**12a**	Definition of and rationale for test positivity cut-offs or result categories of the index test, distinguishing pre-specified from exploratory	eTable 1
	**12b**	Definition of and rationale for test positivity cut-offs or result categories of the reference standard, distinguishing pre-specified from exploratory	eTable 1
	**13a**	Whether clinical information and reference standard results were available to the performers/readers of the index test	n/a
	**13b**	Whether clinical information and index test results were available to the assessors of the reference standard	n/a
Analysis	**14**	Methods for estimating or comparing measures of diagnostic accuracy	10-11
	**15**	How indeterminate index test or reference standard results were handled	n/a
	**16**	How missing data on the index test and reference standard were handled	10
	**17**	Any analyses of variability in diagnostic accuracy, distinguishing pre-specified from exploratory	n/a
	**18**	Intended sample size and how it was determined	10
**RESULTS**			
Participants	**19**	Flow of participants, using a diagram	Figure 1
	**20**	Baseline demographic and clinical characteristics of participants	Table 1
	**21a**	Distribution of severity of disease in those with the target condition	Table 2
	**21b**	Distribution of alternative diagnoses in those without the target condition	n/a
	**22**	Time interval and any clinical interventions between index test and reference standard	n/a
Test results	**23**	Cross tabulation of the index test results (or their distribution) by the results of the reference standard	Table 3
	**24**	Estimates of diagnostic accuracy and their precision (such as 95% confidence intervals)	Table 3
	**25**	Any adverse events from performing the index test or the reference standard	n/a
**DISCUSSION**	**26**	Study limitations, including sources of potential bias, statistical uncertainty, and generalisability	13-15
	**27**	Implications for practice, including the intended use and clinical role of the index test	13-15
**OTHER INFORMATION**	**28**	Registration number and name of registry	n/a
	**29**	Where the full study protocol can be accessed	n/a
	**30**	Sources of funding and other support; role of funders	17

© 2021 Bonnewell JP et al. *JAMA Network Open*.

eTable 3. Risk score prognostic performance for in-hospital death by proposed cutoffs in HIV-infected participants with febrile illness, northern Tanzania, 2016-2019.

Score and cutoff[a]	Sensitivity, % (95% CI)	Specificity, % (95%CI)	Positive predictive value, % (95%CI)[b]	Negative predictive value, % (95%CI)[b]	Positive likelihood ratio	Negative likelihood ratio	AUROC (95%CI)
**MEWS ≥5**	65.7 (47.8-80.9)	64.4 (56.6-71.7)	28.4 (18.9-39.5)	89.7 (82.8-94.6)	1.85 (1.35-2.53)	0.53 (0.33-0.85)	0.65 (0.56-0.74)
**NEWS ≥5**	88.6 (73.3-96.8)	33.1 (26.0-40.9)	22.1 (15.6-29.9)	93.1 (83.3-98.1)	1.32 (1.13-1.56)	0.35 (0.13-0.89)	0.61 (0.54-0.67)
**NEWS ≥7**	77.1 (59.9-89.6)	58.3 (50.3-65.9)	28.4 (19.6-38.6)	92.2 (85.3-96.6)	1.85 (1.43-2.39)	0.39 (0.21-0.73)	0.68 (0.60-0.76)
**qSOFA ≥1**	94.3 (80.8-99.3)	20.2 (14.4-27.2)	20.2 (14.4-27.2)	94.3 (80.8-99.3)	1.18 (1.06-1.32)	0.28 (0.07-1.12)	0.57 (0.52-0.62)
**qSOFA ≥2**	54.3 (36.6-71.2)	74.2 (66.8-80.8)	31.1 (19.9-44.3)	88.3 (81.7-93.2)	2.11 (1.41-3.14)	0.62 (0.42-0.89)	0.64 (0.55-0.73)
**SIRS ≥2**	88.6 (73.3-96.8)	19.0 (13.3-25.9)	19.0 (13.3-25.9)	88.6 (73.3-96.8)	1.09 (0.95-1.26)	0.60 (0.23-1.59)	0.54 (0.48-0.60)
**UVA ≥5[c]**	62.9 (44.9-78.5)	76.1 (68.8-82.4)	36.1 (24.2-49.4)	90.5 (84.3-94.9)	2.63 (1.81-3.82)	0.49 (0.32-0.76)	0.70 (0.61-0.78)

[a]Abbreviations: 95%CI is 95% confidence interval; AUROC is area under the receiver-operator characteristic curve; MEWS is Modified Early Warning Score; NEWS is National Early Warning Score; qSOFA is quick Sequential Organ Failure Assessment; SIRS is Systemic Inflammatory Response Syndrome; UVA is Universal Vital Assessment.
[b]Calculated using an in-hospital death outcome prevalence of 18.0% (95%CI 13.0-23.7), as noted in the HIV-infected cohort in this study.
[c]No HIV-infected participant had a UVA score <2 due to the inclusion of HIV in the calculation of UVA. Thus UVA cutoff ≥2 was not assessed.

eTable 4. Risk score prognostic performance by all ordinal values for in-hospital death in HIV-infected participants admitted with febrile illness, northern Tanzania, 2016-2019.

Prognostic score[a]	AUROC	95%CI
**MEWS**	0.70	0.61-0.80
**NEWS**	0.71	0.62-0.81
**qSOFA**	0.66	0.57-0.75
**SIRS**	0.54	0.44-0.64
**UVA**	0.76	0.67-0.85

[a]Abbreviations: 95%CI is 95% confidence interval; AUROC is area under the receiver-operator characteristic curve; MEWS is Modified Early Warning Score; NEWS is National Early Warning Score; qSOFA is quick Sequential Organ Failure Assessment; SIRS is Systemic Inflammatory Response Syndrome; UVA is Universal Vital Assessment.

© 2021 Bonnewell JP et al. *JAMA Network Open*.
